# Shc3 promotes hepatocellular carcinoma stemness and drug resistance by interacting with β-catenin to inhibit its ubiquitin degradation pathway

**DOI:** 10.1038/s41419-021-03560-8

**Published:** 2021-03-15

**Authors:** Yun Liu, Hao Zhuang, Fang Cao, Jie Li, Yan Guo, Jun Zhang, Qiang Zhao, Yuanyuan Liu

**Affiliations:** 1 Department of Pediatric Oncology, Tianjin Medical University Cancer Institute and Hospital, National Clinical Research Center for Cancer, Key Laboratory of Cancer Prevention and Therapy, Tianjin’s Clinical Research Center for Cancer; Department of Genetics, School of Basic Medical Sciences, Tianjin Medical University, Tianjin, China; 2 Department of Hepatic Biliary Pancreatic Surgery, Cancer Hospital Affiliated to Zhengzhou University, Zhengzhou, Henan Province China; 3 Department of Thoracic Surgery, The Second Hospital of Tianjin Medical University, Tianjin Medical University, Tianjin, China

**Keywords:** Liver cancer, Ubiquitylation

## Abstract

Hepatocellular carcinoma (HCC) is one of the most common cancers with an insidious onset, strong invasiveness, insensitivity to chemotherapy, and poor prognosis, thus makes clinical treatment challenging. The mechanisms require further elucidation for developing novel therapies and targeting drug resistance. Here, we observed high Shc3 expression in patients with chemoresistant and recurrent HCCs. Shc3 overexpression induced a significant increase in MDR1/P-glycoprotein expression, whereas Shc3 knockdown impaired this expression. Further, Shc3 inhibition significantly restored HCC cell sensitivity to doxorubicin and sorafenib. Mechanistically, Shc3 interacted with β-catenin, inhibited destruction complex stability, promoted β-catenin release, and dampened β-catenin ubiquitination. Shc3 bound β-catenin and facilitated its nuclear translocation, prompting the β-catenin/TCF pathway to elevate MDR1 transcription. β-catenin blockage abolished the discrepancy in drug resistance between Shc3-depleted HCC cells and control cells, which further validating that β-catenin is required for Shc3-mediated liver chemotherapy. We also determined the effect of Shc3 on the sensitivity of HCC to chemotherapy in vivo. Collectively, this study provides a potential strategy to target these pathways concurrently with systemic chemotherapy that can improve the clinical treatment of HCC.

## Introduction

Hepatocellular carcinoma (HCC) is one of the most aggressive and common human malignancies globally^1^. Currently, the most commonly used clinical treatment for HCC include percutaneous ablation, curative resection, liver transplantation, transarterial chemoembolization (TACE), and systemic targeted agent like sorafenib. However, most patients are diagnosed at an advanced stage and are not eligible for curative surgery. TACE is the standard of care for patients with unresectable HCC; however, standard chemotherapy has not been found to be beneficial in prolonging the overall survival of HCC patients as it is often associated with substantial toxicity and acquired multidrug resistance^2^. The cause of multidrug resistance (MDR) in cancer is multifaceted, with MDR1 (known as P-glycoprotein, ABCB1) overexpression being the key determinant of HCC drug resistance^3,4^. Inherent high resistance of HCC to chemotherapeutic drugs and early HCC recurrence are the main causes of HCC related death. Accumulating research findings has shown that recurrence and chemoresistance of HCC are closely related to HCC stemness, and that the epithelial-mesenchymal transition (EMT) program also appears to contribute to liver cancer stem cells (CSCs) phenotype through its effects on the intracellular signaling pathway^5^. For these reasons, identification of the key molecules involved in multidrug resistance of HCC is imperative for improving the clinical outcome.

Src homolog and collagen homolog 3 (Shc3) is an adaptor protein belonging to the Shc family. The *SHC3* (known as Rai, N-Shc, ShcC) gene encodes for two isoforms, p52Shc3 and p64Shc3; all isoforms have conserved domains with an PTB-CH1-SH2 domain modular structure and p64Shc3 has an additional N-terminal CH2 domain^6^. Shc3 has been implicated in cell survival and differentiation; the downregulation of Shc3 could induce abnormal alterations in hypoxic signaling, apoptosis, and inflammatory response^7^. Recently, several studies on Shc3 in malignant tumors have been conducted. Shc3 is ectopically overexpressed in various cancers, such as high-grade astrocytomas, high-grade glioblastomas^8^, neuroblastomas^9^, thyroid carcinoma^10^, and hepatocellular carcinomas^11^. Shc3 interacts with Gab1 and recruits the p85 subunit of PI3K, which leads to downstream activation of the Akt pathway in papillary thyroid carcinoma. In neuroblastoma cells, the interplay between Shc3 and HIF-1α may protect of cancer cells against hypoxia. Also studies have reported that Shc3 is a new regulator of cancer stem cell migration, and Shc3 silencing in glioblastoma can reduce migration and invasion^10,12,13^. Our previous study reported that Shc3 forms a complex with MVP, MEK, and ERK to potentiate ERK activation independent of c-Raf. This interaction consequently induces EMT and promotes HCC cell metastasis, thus we speculate that Shc3 may plays an important role in sorafenib resistance in HCC^11^. Moreover, recent reports have indicated that EMT induction in tumor cells not only contributes to increased metastasis, but also leads to MDR, and we presume that Shc3 is associated with drug sensitivity. However, little is known about the molecular mechanism interplay Shc3 with MDR.

β-catenin/T-cell factor signaling plays a central role in carcinogenesis by regulating cell differentiation, proliferation, metastasis, drug resistance, and stemness^14,15^. Ectopic activation of the β-catenin pathway has been found in a wide range of tumors of intestinal, liver and hematopoietic cell origin^16^. β-catenin is the key molecule in this pathway, and its protein levels and nuclear translocation are tightly controlled by the multiprotein β-catenin destruction complex. Cytoplasmic β-catenin is constitutively degradated by two scaffolding proteins, adenomatous polyposis coli (APC) and Axin, which requires scaffolding the Ser/Thr kinases glycogen synthase kinase 3 (GSK-3), casein kinase 1 (CK1) and β-catenin to facilitate the amino terminus phosphorylation of β-catenin. Phosphorylated β-catenin is recognized by ubiquitin ligase β-transducin repeat-containing protein (β-TrCP), and is subsequently targeted for ubiquitin-mediated proteasomal degradation^15^. Disassembly of the destruction complex can block β-catenin degradation that results in the translocation of the accumulated β-catenin into the nucleus, where β-catenin binds to the lymphoid enhancer factor/T-cell factor (LEF/TCF) family of transcription factors and triggers the protooncogene-induced transcription of several target genes, such as c-myc, MDR1, OCT4, and cyclin D1 (refs. ^17–19^). Hyper-activation of the β-catenin/TCF signal is frequently detected in human HCCs^20^ and high nuclear expression of β-catenin correlated with reduced recurrence-free survival and vascular invasion, suggesting the β-catenin activation are involved in the promotion of HCC recurrence^21^. Hence, a better understanding of the mechanisms underlying the activation of β-catenin/TCF signaling would increase HCC therapeutic benefit.

In this study, we investigated the function of Shc3 in HCC recurrence and drug resistance. We firstly observed high expression of Shc3 in both MDR1 overexpression and recurrent HCCs from patients. Then we verified the effect of Shc3 expression on the stemness and drug resistance by cell function experiments. Mechanistically, Shc3 interacted with the β-catenin, promoted β-catenin release from the destruction complex and dampened the ubiquitination of β-catenin. Consistently, Shc3 facilitated the nuclear translocation of β-catenin and activated MDR1 expression in HCC cells via the β-catenin/TCF-dependent pathway. Our study has uncovered a new mechanism between Shc3 and MDR1 expression, which emphasizes the importance of the β-catenin/TCF pathway in the regulation of drug resistance of HCC.

## Results

### Shc3 is a critical oncogene linked to cancer drug resistant in HCC

**Fig. 1:**
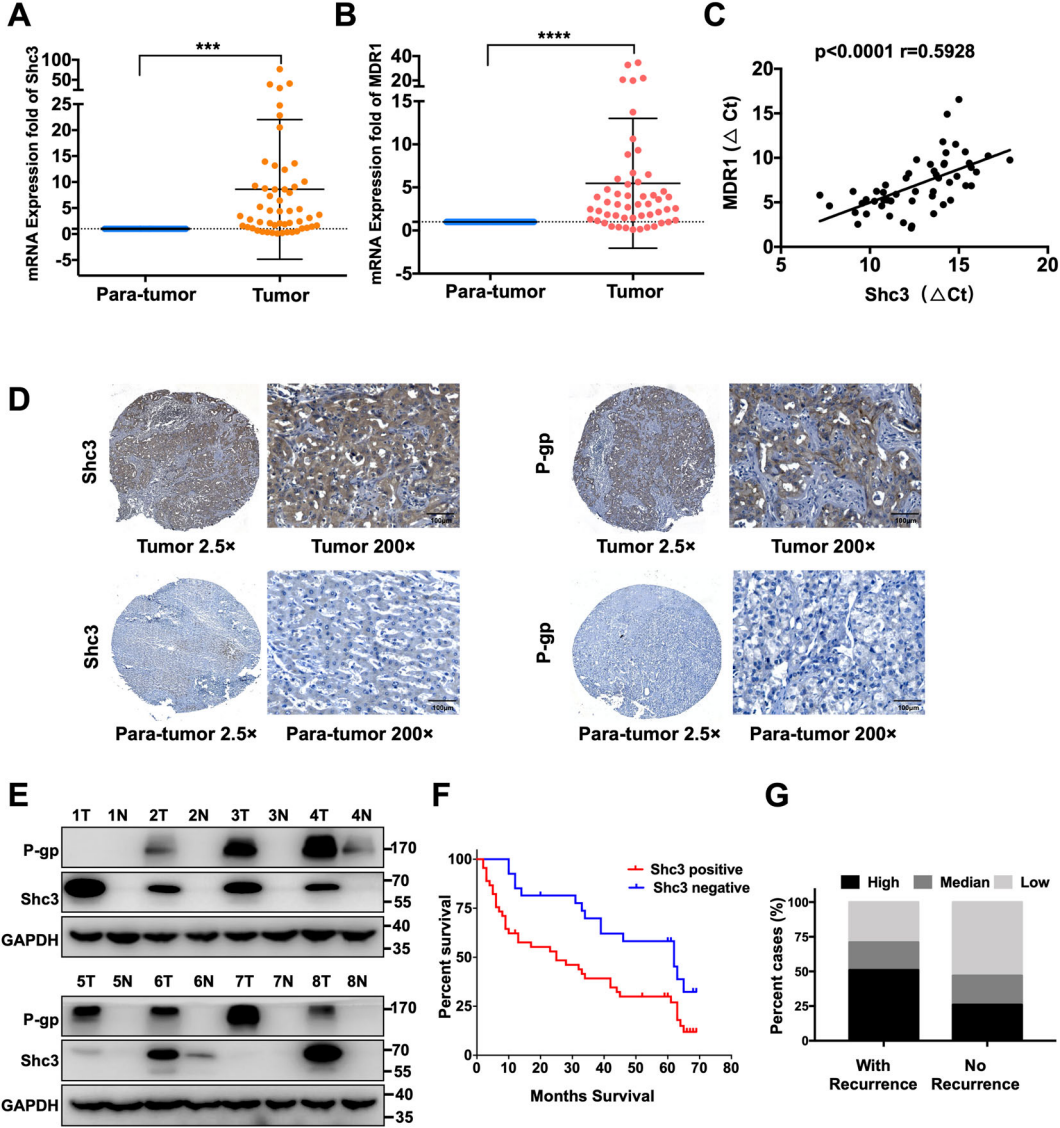
Shc3 is a critical oncogene linked to hepatocellular carcinoma (HCC) drug resistance. **A** The mRNA levels of Shc3 in 52 paired HCC tissues. Shc3 mRNA expression in HCC clinical samples and para-tumor samples were compared using paired Student’s *t*-test, ***P < 0.001. **B** The mRNA levels of MDR1 in 52 paired HCC clinical tissues. Data represent the mean ± SD (****P < 0.0001, by Student’s *t*-test). **C** The correlation between Shc3 and MDR1 expressions in 52 paired HCC samples (Pearson’s correlation coefficient, *R* = 0.5928, *P* < 0.0001). **D** Representative immunohistochemistry analysis of Shc3 and P-gp expression in 72 paired HCC tissues (magnifications: ×2.5 and ×200). **E** Western blot analysis of Shc3 and P-gp expression in 8 pairs of HCC and non-tumor liver tissues. T, HCC tissues; N, non-tumor liver tissues. **F** Kaplan–Meier’s correlation analyses between Shc3 expression levels and overall survival. **G** According to the IHC staining intensity, the expression levels of Shc3 in 72 HCC patients were classified as low, medium, and high. The correlation of Shc3 expression and HCC recurrence was examined.

Our previous study has shown that aberrant expression of Shc3 may play an important role in sorafenib resistance in HCC, thus we are wondering that whether Shc3 is involved in HCC multidrug resistance. To determine the relationship between Shc3 and multidrug resistance, the clinical relevance of Shc3 and MDR1 messenger RNA (mRNA) expression level in 52 matched HCC and non-tumor liver tissues was examined by quantitative reverse-transcription PCR (qRT-PCR). We observed a dramatically higher expression of Shc3 in HCC tumor than in the para-tumors (82.69%, 43 of 52); higher MDR1 transcript levels were observed in 80.76% (42 of 52) of HCC samples than its corresponding para-tumor tissues (Fig. 1A, B and Table S1). The mRNA levels of Shc3 and MDR1 in HCC tumor tissues and adjacent para-tumor samples were compared. Notably, we found that the Shc3 transcript level was positively associated with MDR1 mRNA level (Fig. 1C). Thereafter, we examined Shc3 and P-gp protein expression in paraffin-embedded HCC tissue samples and adjacent para-tumor samples from 72 additional patients using IHC. The results showed that Shc3 and P-gp were upregulated in HCC tissues (Fig. 1D). We also detected the protein expression levels of Shc3 and P-gp in tissue samples from eight HCC patients. Western blot analysis showed that Shc3 and P-gp expression was consistent with the IHC results (Fig. 1E). These results suggested that both Shc3 and P-gp expression are high in HCC tissues, and an association between Shc3 and MDR1 expression might exist.

We further analyzed the association between Shc3 protein expression and clinicopathological characteristics in 72 HCC patients. Higher expression of Shc3 was detected in 46 of the 72 samples when expression of Shc3 was compared with that of the corresponding non-tumor liver tissues (Table 1), and Shc3 protein expression was similar to P-gp protein expression in HCC samples (*χ*^2^ = 7.127, *P* = 0.007). Furthermore, expression of Shc3 was associated with maximal tumor size (*χ*^2^ = 5.471, *P* = 0.018), microvascular invasion (*χ*^2^ = 7.799, *P* = 0.005), malignant differentiation (*χ*^2^ = 6.981, *P* = 0.008), TNM stage (*χ*^2^ = 7.472, *P* = 0.005; Table 1), and Kaplan–Meier analysis showed that HCC patients with high Shc3 levels displayed shorter overall survival (*χ*^2^ = 6.847, *P* = 0.009; Fig. 1F). Notably, clinical investigations demonstrated that patients with high Shc3 levels possessed a higher risk of HCC recurrence (Fig. 1G). The above results suggested that Shc3 expression is a crucial independent prognostic factor that potentially plays a crucial role in HCC recurrence and drug resistance.

**Table 1:** Clinicopathological information of hepatocellular carcinoma patients.

	All	Shc3 expression		*P -*value
		Positive (*n* = 46)	Negative (*n* = 26)	
Gender				0.521
Male	62	40	22	
Female	10	6	4	
Age				0.170
≤60 years	48	33	15	
>60 years	24	13	11	
Serum AFP level (ng/mL)				0.410
≤400	50	31	19	
>400	22	15	7	
HBsAg				0.310
Positive	64	42	22	
Negative	8	4	4	
Cirrhosis				0.283
Present	54	36	18	
Absent	18	10	8	
Maximal tumor size				0.018*
≤5 cm	26	12	14	
>5 cm	46	34	12	
Tumor number				0.392
Single	64	40	24	
Multiple	8	6	2	
Microvascular invasion				0.005*
Present	38	30	8	
Absent	34	16	18	
Macrovascular invasion				0.093
Present	9	8	1	
Absent	63	38	25	
Tumor differentiation				0.008*
High grade (I/II)	27	12	15	
Low grade (III/IV)	45	34	11	
Tumor encapsulation				0.111
Present	60	36	24	
Absent	12	10	2	
TNM staging				0.005*
Early stage (I/II)	46	24	22	
Advanced stage (III/IV)	26	22	4	
P-gp expression				0.007*
Positive	40	31	9	
Negative	32	15	17	

*AFP* alpha fetal protein, *HBsAg* hepatitis B surface antigen.

*P < 0.05.

### Identification of Shc3 as a positive regulator of MDR1/P-gp expression

**Fig. 2:**
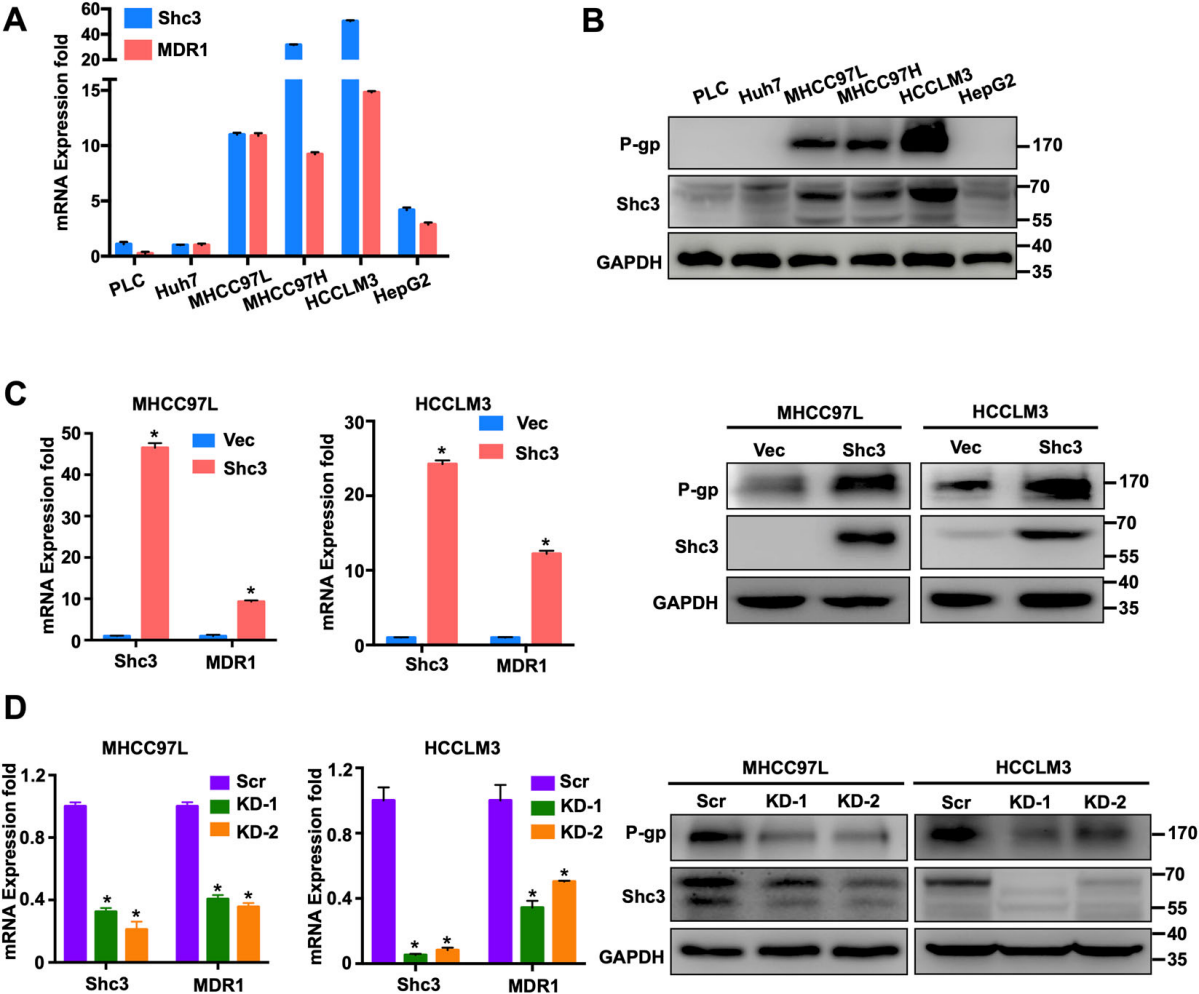
Identification of Shc3 as a positive regulator of MDR1/P-gp expression. **A** The mRNA levels of Shc3 and MDR1 in six liver cancer cell lines were analyzed by qRT-PCR. **B** Shc3 and P-gp protein expression in six liver cancer cell lines. **C** Overexpression of Shc3 resulted in increased expression of MDR1 in MHCC97L and MHCCLM3 cells. (left) Relative mRNA expression of Shc3 and MDR1 were examined by qRT-PCR; (right) Relative protein expression levels of Shc3 and P-gp were detected by Western blotting. **D** Knockdown of Shc3 reduced the expression of MDR1 in MHCC97L and MHCCLM3 cells. Shc3 expression was knocked down by short hairpin RNAs. Data represent the mean ± SD (*P < 0.05 vs. control, by one-way ANOVA or Student’s *t*-test).

To further investigate the relationship between Shc3 and MDR1, we examined the expression levels of Shc3 and MDR1/P-gp in numerous HCC cells. As anticipated, the mRNA level of Shc3 significantly increased in malignant and P-gp high-expression MHCC97L, MHCC97H, and HCCLM3 cells (Fig. 2A), and western blot results confirmed this increased in the malignant and P-gp high-expression cells (Fig. 2B). Meanwhile, the qRT-PCR results revealed that Shc3 expression correlated with MDR1 in HCC cells (Fig. S1, Pearson correlation coefficient *R* = 0.7596, *P* < 0.05). As MDR1 overexpression is the key determinant of HCC drug resistance, so we further detected the effects of Shc3 on the expression of MDR1. As shown in Fig. 2C, D, stable Shc3 overexpression resulted in significantly increased MDR1 expression in both MHCC97L and HCCLM3 cells, as examined by qRT-PCR and western blot analyses. Meanwhile, stable depletion of Shc3 also resulted in remarkable decrease of the expression of MDR1. Taken together, these results further verified the role of Shc3 in promoting expression of multidrug resistance genes and that MDR1 is likely to be a downstream target gene of Shc3.

### Inhibition of Shc3 expression restores drug sensitivity of HCC cells

**Fig. 3:**
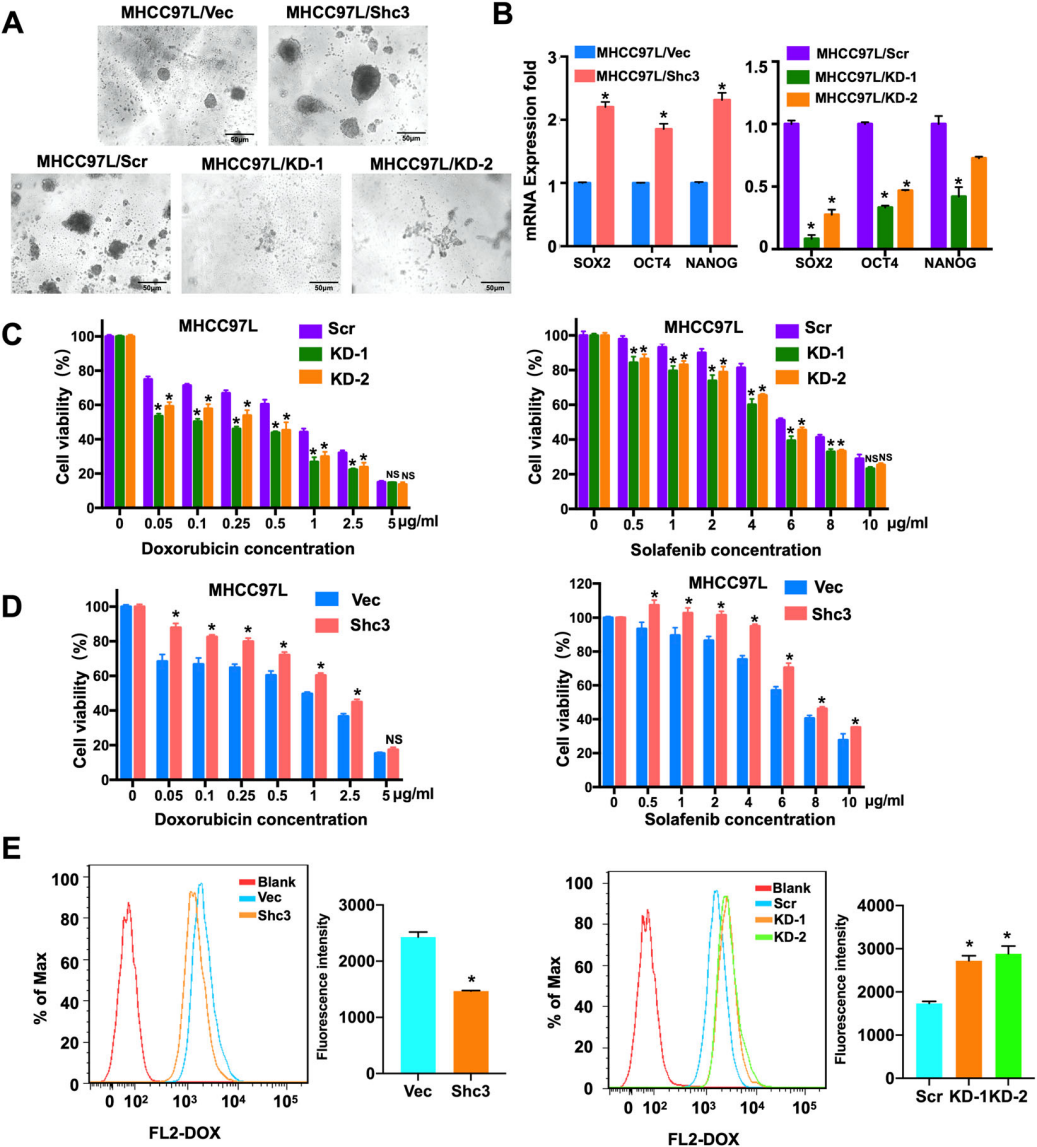
Inhibition of Shc3 expression restores chemotherapeutic drug sensitivity of HCC cells. **A** Spheroid formation assay of MHCC97L/Shc3 or MHCC97L/KD and their control cells. A representative image is shown. **B** MHCC97L cells infected by lentivirus expressing Shc3 or shShc3 were collected and relative mRNA expression of SOX2, OCT4, and NANOG were examined by qRT-PCR. **C** Shc3 knockdown remarkably restored sensitivity of MHCC97L cells to doxorubicin (left) and sorafenib (right) in a dose-dependent manner. MHCC97L/KD or MHCC97L/Shc3 and their control cells were treated with drugs for 48 h. Cell viability was calculated using the CCK8 assay and compared with 100% viability non-treated cells. Data are shown as mean ± SD; *n* = 5; *P < 0.05 compared with control. **D** Overexpression of Shc3 significantly promoted drug resistance of MHCC97L cells to doxorubicin (left) and sorafenib (right). Data are shown as mean ± SD; *n* = 5; *P < 0.05 compared with control. **E** Flow cytometry analysis of MHCC97L/Shc3 (left) and MHCC97L/KD cells (right) after 4 h incubation with free doxorubicin. The comparison of intracellular fluorescence intensities of different groups were shown. Data are shown as mean ± SD; *n* = 3. Error bars represent the mean ± SD (*P < 0.05 vs. control, by one-way ANOVA or Student’s *t*-test).

Considering the close association of HCC stemness with HCC recurrence and chemoresistance, we examined the functional role of Shc3 in HCC stemness. Sphere formation ability is indicative of the self-renewal and pluripotent properties of CSCs^22^. Results of the spheroid formation assay shown that stable Shc3 overexpressed MHCC97L cells formed more spheroids than MHCC97L/Vector cells (Fig. 3A, up). In addition, downregulation of Shc3 expression led to the formation of fewer spheroids than control cells (Fig. 3A, down), which further indicates that Shc3 inhibition decreased the proportion of liver CSCs. To address the effect of Shc3 on cellular stemness-related molecules, we evaluated the expression of several pluripotency transcription factors (SOX2, OCT4 and NANOG) in Shc3 overexpression and knockdown cells. qRT-PCR analysis results showed that Shc3 overexpression had higher expression levels of SOX2, OCT4, NANOG than the control cells and knockdown of Shc3 reduced the stem cell-related transcription factors (Fig. 3B). All these results indicated that Shc3 promotes tumor stemness.

Owing to the previous results demonstrating that Shc3 promotes MDR1 expression, we hypothesized that increased Shc3 expression can contribute to promote drug resistance of HCC cells. Thus, we analyzed the effect of Shc3 expression on cell sensitivity to sorafenib and doxorubicin by the Cell Counting Kit-8 (CCK8) assay. As shown in Fig. 3C, D, depletion of Shc3 expression remarkably increased the sensitivity of MHCC97L cells to sorafenib and doxorubicin, whereas Shc3 overexpression enhanced cell drug resistance in MHCC97L cells. Similar results were observed in HCCLM3 cells (Fig. S2A), further confirming the drug resistant ability of Shc3. P-gp is an important membrane protein that has the ability to pump numerous foreign substances out of cells, and several studies have shown that P-gp is overexpressed in HCC, which may lead to chemotherapy failure by decreasing the intracellular accumulation of antitumor agents^23^. To investigate the effect of Shc3 on drug efflux, we examined the cellular uptake of doxorubicin by flow cytometry. The results demonstrated that Shc3 knockdown cells notably elevated cellular uptake of doxorubicin compared with the control cells (Fig. 3E, right). Meanwhile, the intracellular accumulation of doxorubicin was decreased when overexpress Shc3 (Fig. 3E, left). Taken together, above results demonstrated that Shc3 increased drug resistance of HCC cells to chemotherapeutic agents.

### Shc3 promotes β-catenin release from the destruction complex

**Fig. 4:**
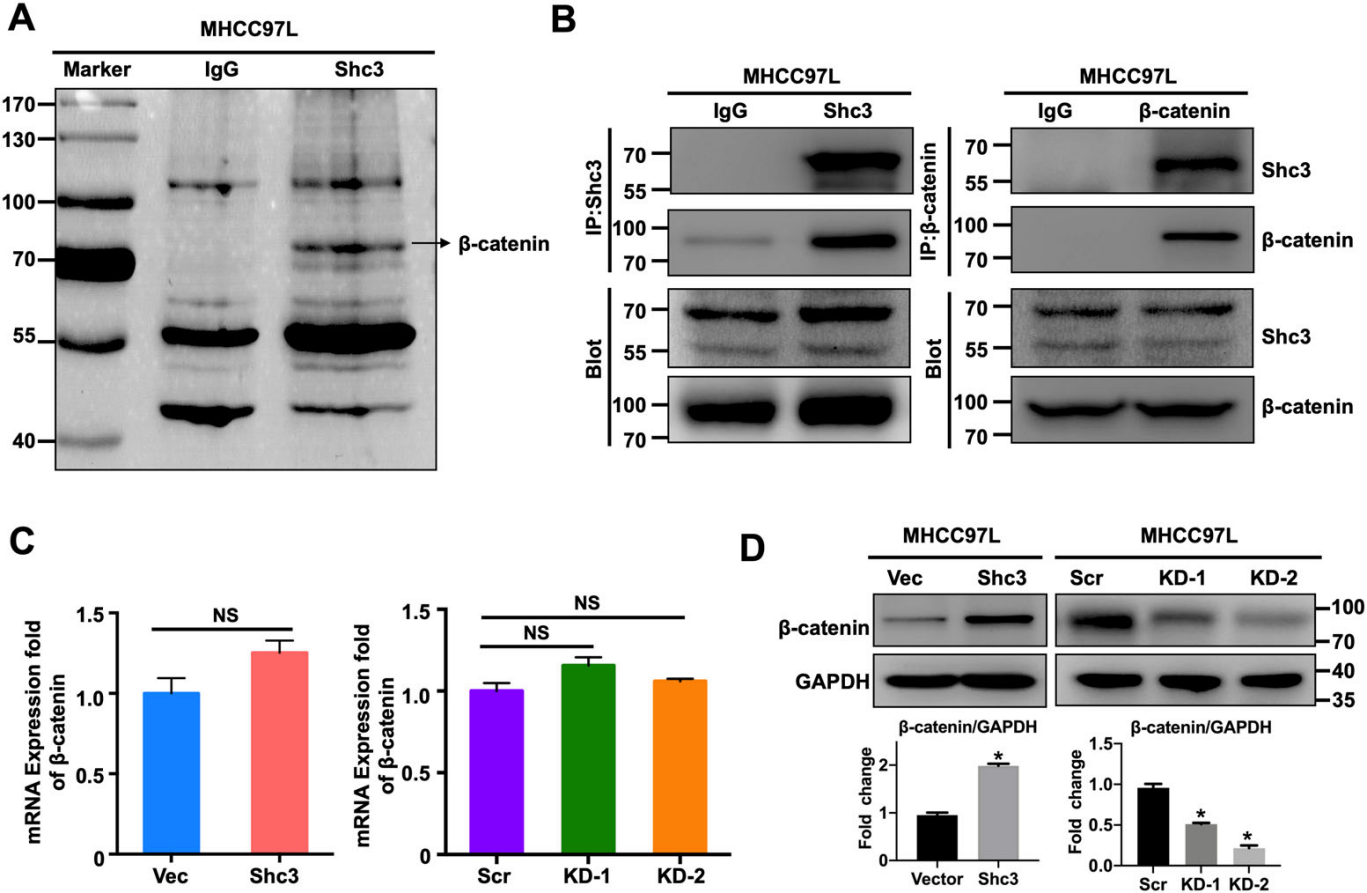
Shc3 and β-catenin interaction potentiates β-catenin signaling activation. **A** Image of a silver stained SDS–PAGE gel. IP was performed with MHCC97L cell lysates. Normal IgG was used as a control. **B** Co-IP of endogenous Shc3 interacts with β-catenin in MHCC97L cells. **C**, **D** qRT-PCR and western blot analysis of β-catenin mRNA and protein expression levels in MHCC97L Shc3-overexpressing and Shc3-knockdown cells. Right bottom, the histogram shows the comparison of the relative intensity of β-catenin and GAPDH. Error bars represent the mean ± SD (*P < 0.05 vs. control, by one-way ANOVA or Student’s *t*-test).

We next sought to understand the mechanisms by which Shc3 promotes cancer cell stemness and drug resistance in HCC. Immunoprecipitation and mass spectrometry were carried out to identify proteins that interact with Shc3. Mass spectrometry results showed that β-catenin associates with Shc3 (Fig. 4A and Fig. S3A). Then coimmunoprecipitation experiments were conducted to assess the interaction between Shc3 and β-catenin using MHCC97L cell extracts; the results confirmed this interaction (Fig. 4B). To further understand the functional association between Shc3 and β-catenin, we examined the mRNA levels of β-catenin by qRT-PCR assays. Unexpectedly, neither Shc3 overexpression nor Shc3 knockdown altered the mRNA levels of β-catenin (Fig. 4C). However, western blotting showed that overexpression of Shc3 significantly increase β-catenin expression, whereas Shc3 knockdown impaired β-catenin expression (Fig. 4D and Fig. S3B), suggesting Shc3 might regulate β-catenin protein levels via a post-transcriptional mechanism. Collectively, these results suggested that β-catenin activity is likely mediated by Shc3.

**Fig. 5:**
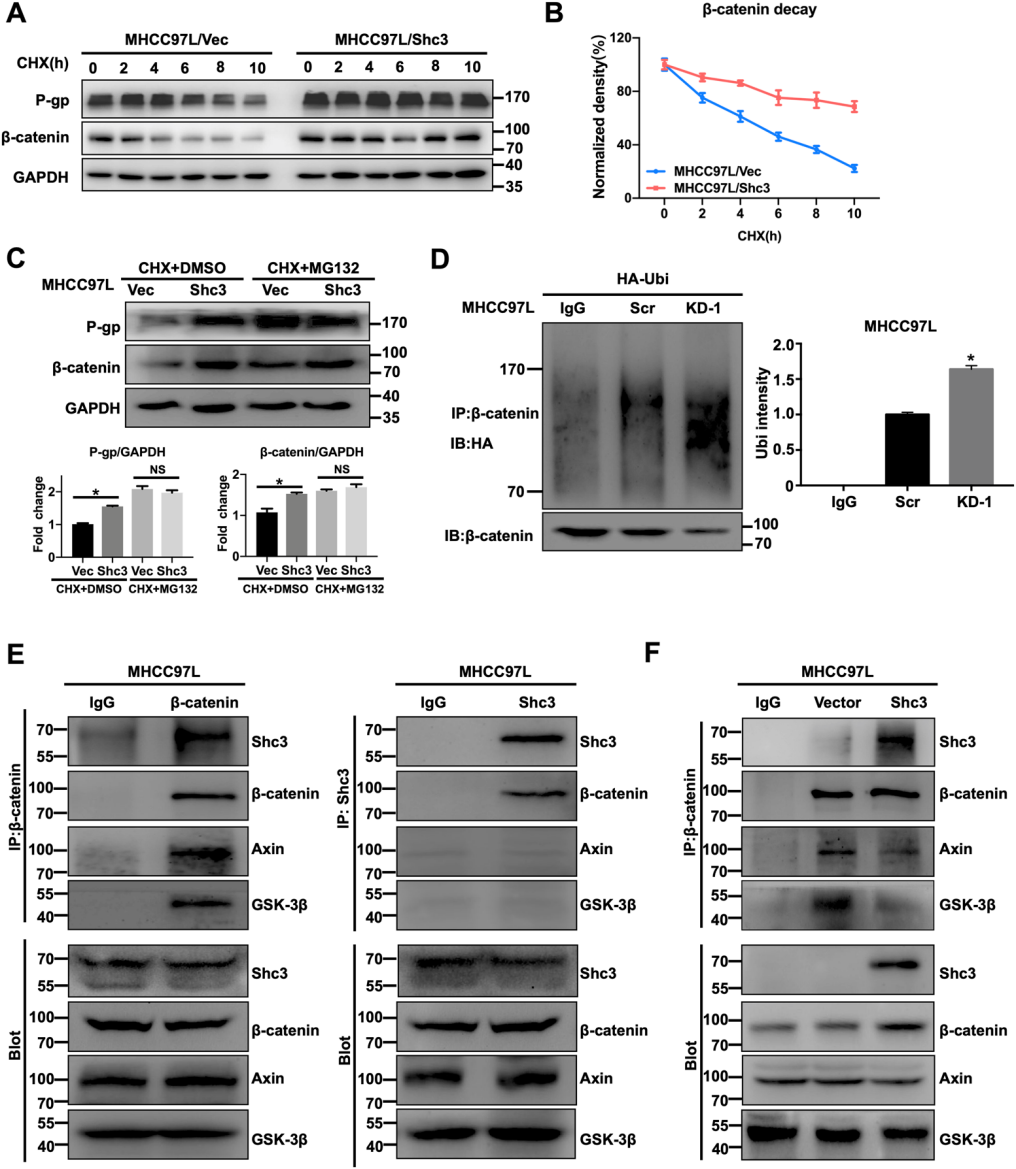
Shc3 promotes β-catenin release from the destruction complex. **A** Shc3 overexpressed cells and its control cells were treated with CHX (10 μg/mL), and total proteins were collected at the indicated time points. The protein expression levels of β-catenin and P-gp were examined by western blot assay. **B** The line graph shows the comparison of the relative intensity of β-catenin and GAPDH through densitometric analysis. **C** MHCC97L/Shc3 and MHCC97L/Vector cells were incubated with CHX (10 μg/mL) either with or without MG132 (10 μM). Up, Western blotting was used to assess the protein expression levels of β-catenin and P-gp. Bottom, the histogram shows the comparison of the relative intensity of P-gp and GAPDH, β-catenin and GAPDH. **D** Following transfection with the HA-tagged ubiquitin plasmid, MHCC97L/KD-1 cells and MHCC97L/Scr cells were lysised and immunoprecipitated with anti-β-catenin antibody and then detected by western blotting with anti-HA or anti-β-catenin (left). Error bars represent the mean ± SD (*P < 0.05 MHCC97L/KD-1 vs. MHCC97L/Scr, by Student’s *t*-test). **E** Coimmunoprecipitation of β-catenin, Shc3, Axin, and GSK-3β, which were pulled down using anti-β-catenin or anti-Shc3 in MHCC97L cells. **F** Co-IP analysis of destruction complex proteins (β-catenin, Axin, GSK-3β) and Shc3, which were pulled down using anti-β-catenin or an IgG antibody in MHCC97L/Shc3 and MHCC97L/Vector cells.

β-catenin is a key regulator in the Wnt pathway, and is primarily distributed in the cytoplasm and degraded by ubiquitin-mediated protease^24^. Thus, we speculated that Shc3 might regulate β-catenin protein stability. To define the underlying molecular mechanism, we firstly tested the degradation of β-catenin in the presence of cycloheximide (CHX), a potent inhibitor of protein translation. As CHX (10 μg/mL) treatment time was prolonged, degradation of β-catenin increased, and the degradation was impaired when Shc3 was overexpressed, suggesting that Shc3 might be involved in β-catenin degradation (Fig. 5A, B). In addition, treatment with 10 μM MG132, a proteasomal inhibitor, salvaged Shc3-induced in β-catenin levels increases, further conforming the role of Shc3 in β-catenin degradation (Fig. 5C). These results suggested that the ubiquitination degradation pathway was involved, and the proteasome-mediated degradation of ubiquitinated proteins is a main pathway to regulate the expression of various proteins in cells. Then the ubiquitylation of β-catenin was further detected by western blotting. The results showed that decreased endogenous β-catenin ubiquitination in the Shc3-overexpressed cells compared with the corresponding control cells (Fig. S4), whereas Shc3 knockdown promoted endogenous β-catenin ubiquitination in the Shc3-knockdown cells (Fig. 5D).

To further verify this regulatory mechanism and determine whether Shc3 inhibits destruction complex stability and promotes β-catenin release from the destruction complex. We conducted endogenous IP assays using the anti-β-catenin antibody. β-catenin coimmunoprecipitated with Shc3 and destruction complex protein Axin and GSK-3β in MHCC97L cells. Surprisingly, IP with Shc3 antibody pulled down β-catenin but not Axin and GSK-3β in MHCC97L cells. Furthermore, as shown in Fig. 5F, Shc3 overexpression inhibited the interaction between the destruction complex, including GSK-3β, Axin and β-catenin, suggesting that Shc3 may compete with destruction complex for β-catenin binding. These results indicated that Shc3 overexpression inhibits β-catenin ubiquitin-proteasome pathway degradation by inhibiting the interaction between the destruction complex, including GSK-3β, Axin, and β-catenin.

### Shc3 activates MDR1 expression in HCC cells through β-catenin translocation into the nucleus

**Fig. 6:**
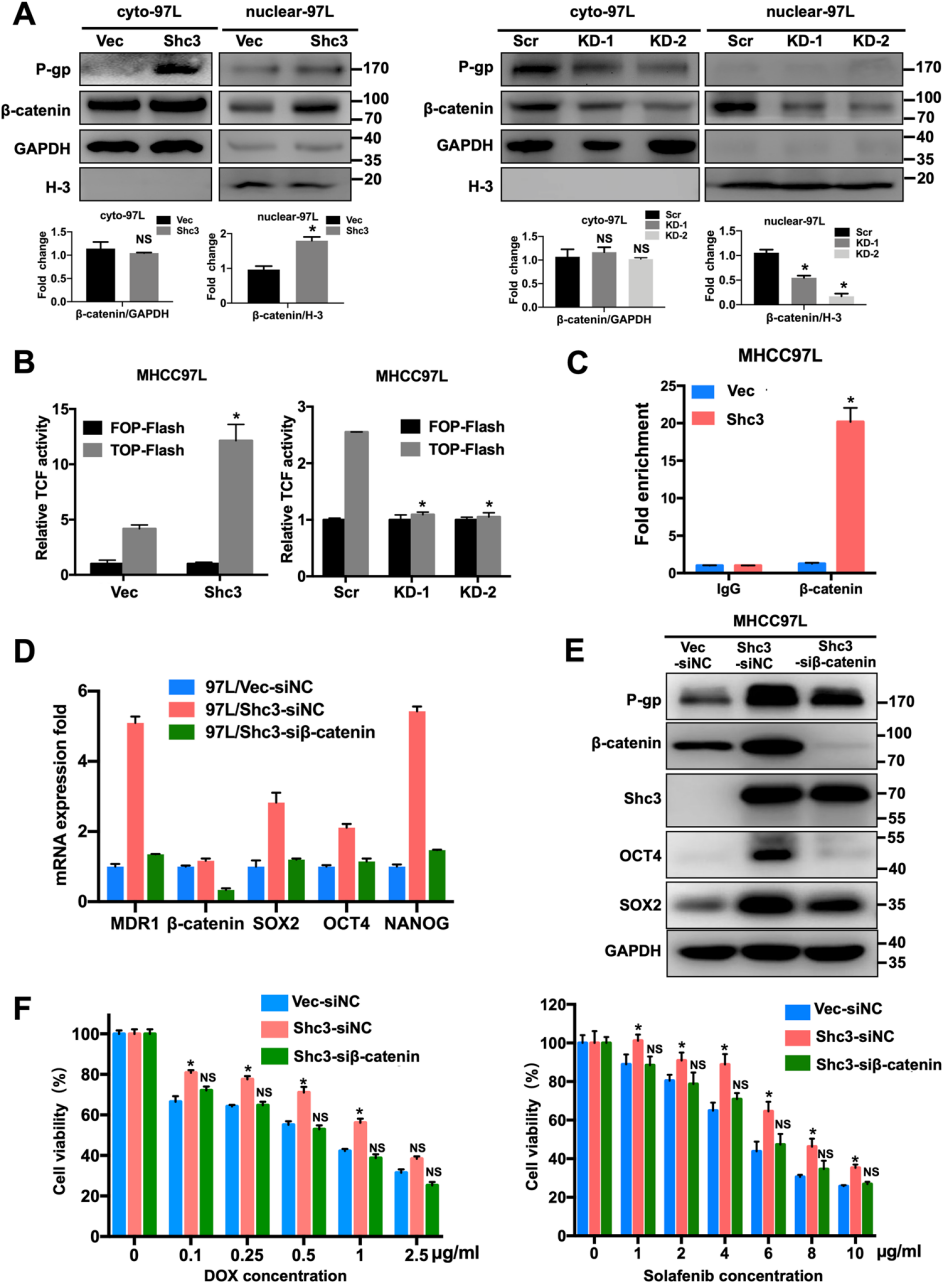
Shc3 induces MDR1 expression in HCC cells via β-catenin signaling pathway. **A** Up, the relative nuclear and cytoplasmic expression of P-gp, β-catenin and Shc3 was compared between MHCC97L/Shc3 or MHCC97L/KD cells and its control cells using western blots. Bottom, the histogram shows the comparison of the relative intensity of β-catenin and GAPDH, β-catenin and H-3. **B** Changes in β-catenin/TCF reporter activity in overexpression or knockdown Shc3 MHCC97L cell lines, Data are shown as mean ± SD; *n* = 3; *P < 0.05. **C** ChIP-PCR experiment of β-catenin binding to the promoter of MDR1 in MHCC97L/Vector and MHCC97L/Shc3 cells, Data are shown as mean ± SD; *n* = 3; *P < 0.05. **D**, **E** β-catenin siRNAs were transfected into Shc3-overexpressing MHCC97L cells. qRT-PCR was used to examine the relative mRNA expression levels of MDR1, β-catenin, SOX2, OCT4 and NANOG; western blot analysis was used to examine the relative expression levels of P-gp, β-catenin, Shc3, OCT4 and SOX2. **F** Shc3-overexpressing MHCC97L cells transfected with β-catenin siRNAs were treated with doxorubicin (left) and sorafenib (right) for 48 h. Data are shown as mean ± SD; *n* = 5; *P < 0.05. Cell viability was calculated using the CCK8 assay and compared with 100% viability non-treated cells. Error bars represent the mean ± SD (*P < 0.05 vs control, by one-way ANOVA or Student’s *t*-test).

Thereafter, we aimed to elucidate how Shc3 affects MDR1 expression by regulating β-catenin translocation. We performed western blot assay, which revealed that the nuclear accumulation of β-catenin evidently enhanced in MHCC97L/Shc3 cells compared with MHCC97L/Vector cells (Fig. 6A, left). Consistently, nuclear translocation of β-catenin was significantly reduced in Shc3-deleted cells (Fig. 6A, right). In addition, confocal laser scanning microscope (CLSM) was employed to visually display intracellular releases of doxorubicin and nuclear translocation of β-catenin in MHCC97L/Scr and MHCC97L/KD cells. As expected, confocal imaging results revealed strong doxorubicin fluorescence in MHCC97L/KD cells, while MHCC97L/Scr cells showed much weaker doxorubicin fluorescence and stronger nuclear translocation of β-catenin (Fig. S5).

To address whether Shc3 enhances β-catenin/TCF activity in HCC cells, we carried out β-catenin/TCF-dependent luciferase assays in MHCC97L cells. The TOPFlash reporter assay showed that much higher β-catenin transcriptional activities in Shc3 overexpressed cells than vector cells; when Shc3 was depleted in MHCC97L cells, the TOPFlash assay revealed that dramatically reduced β-catenin transcriptional activity (Fig. 6B). Previous investigations have demonstrated that activation of the β-catenin/TCF pathway promotes MDR1 expression in chemoresistant neuroblastoma cells and colorectal cancer^25,26^. In addition, the above results demonstrated that this pathway is involved in the activation of MDR1 in HCC cells; thus, we identified β-catenin as a predicted transcription factor (TF) that binds to the MDR1 promoter region in HCC cells. In chromatin-immunoprecipitation (ChIP) assays, the MDR1 promoter was pulled down in anti-β-catenin IP assays in MHCC97L/Shc3 cells much more than in MHCC97L/Vector cells, suggesting that β-catenin binds to the MDR1 promoter under Shc3 regulation (Fig. 6C).

Based on the above results, we hypothesized that MDR1 activation is dependent on Shc3 regulation, mediated by the β-catenin/TCF pathway. To confirm this hypothesis, we transfected Shc3-overexpressing MHCC97L cells with short interfering RNAs targeting β-catenin (siβ-catenin). qRT-PCR and western blot results revealed that downregulation of β-catenin blocked Shc3-mediated expression of MDR1 and stemness-associated transcription factors (SOX2, OCT4 and NANOG) (Fig. 6D, E). In addition, attenuating β-catenin association restored the cell sensitivity to doxorubicin and sorafenib in a dose-dependent manner in MHCC97L/Shc3 cells (Fig. 6F). Collectively, these results demonstrated that MDR1 is upregulated in β-catenin/TCF pathway activation cells and this activity is likely mediated by Shc3.

### Shc3 overexpression inhibits HCC sensitivity to doxorubicin in vivo

**Fig. 7:**
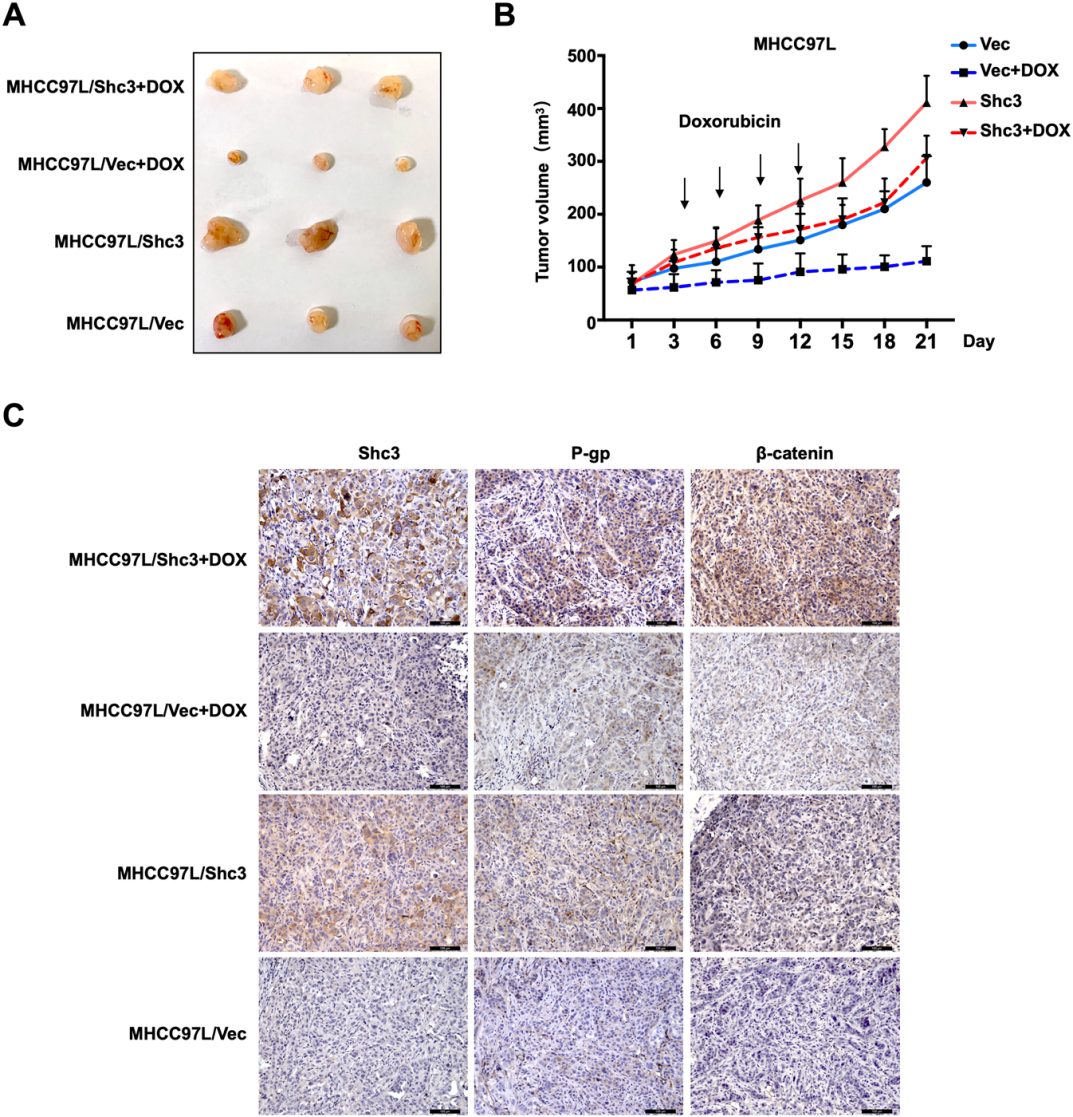
Shc3 overexpression inhibits HCC sensitivity to doxorubicin in vivo. **A** Photographs of tumors removed from different groups of nude mice treated with doxorubicin or PBS. **B** Tumors growth curve of different groups that nude mice with treatment of doxorubicin or PBS. **C** The protein expression levels of Shc3, P-gp and β-catenin were analyzed by IHC assay of tumor tissues from different treatment groups of nude mice (representative images of IHC staining in xenograft models were shown, magnifications: ×200). Data are Error bars represent the mean ± SD.

A nude mouse xenograft model was used to evaluate the functional role of Shc3 in regulating drug resistance of HCC in vivo. Nude mice were subcutaneously inoculated with MHCC97L/Vector or MHCC97L/Shc3 cells (2 × 10^6^ cells per mouse). Ten days later, mice were treated with doxorubicin (20 μg/g) via intravenous injection every other day for four times consecutively, and the tumor volumes were monitored and recorded. Xenograft tumors grew much faster in the MHCC97L/Shc3 group when treated or not treated with doxorubicin compared with the MHCC97L/Vector group (Fig. 7A, B). Correspondingly, IHC staining of the tumor tissues for Shc3 confirmed the efficiency of Shc3 overexpression and revealed the correlation between Shc3 and the levels of P-gp and β-catenin in vivo (Fig. 7C). P-gp and β-catenin were increased in tumor tissues of mice injected with Shc3-overexpressing MHCC97L cells. These results suggested that Shc3 overexpression inhibits the HCC sensitivity to doxorubicin in vivo.

## Discussion

Various targeted and immune therapies have been tested on HCC patients and only sorafenib, a multi-kinase inhibitor, has demonstrated modest efficacy. Moreover, only approximate 20% of HCC patients respond positively to sorafenib treatment^27^. Sorafenib is a potent inhibitor for Raf kinase and several receptor tyrosine kinases, including platelet-derived growth factor receptor (PDGFR), vascular endothelial growth factor receptor 2 (VEGFR-2), c-Kit, and so on. We previously reported that ectopic expression of Shc3 promotes metastasis and proliferation via c-Raf–independent activation of the MEK-ERK pathway; thus, it can be speculated that this alternative pathway regulated by Shc3 may play an critical role in sorafenib resistance^11^. However, the potential molecular mechanism and clinical significance remains unknown and requires further research. In this study, we presented preliminary evidence for a novel mechanism in which Shc3 promotes HCC multidrug resistance.

Our data suggested that Shc3 and P-gp were both upregulated in HCC samples and demonstrated a strong positive correlation. We also found that Shc3 was required for the activation of MDR1 in HCC cells. Moreover, univariate and multivariate analyses showed that elevated Shc3 expression in recurrent HCC. The existence of cancer stem cells is considered the origin of chemoresistance and HCC recurrence^28^. An important feature of CSCs is that they overexpress ABC transporter proteins, which pump many foreign substances including chemotherapeutic drugs out of the cells that may lead to chemotherapy failure. Considering the importance of cancer stem cells in chemoresistance and cancer recurrence, we examined the influence of Shc3 on HCC stemness. We detected more expanded stem cell population in MHCC97L/Shc3 cells compared with MHCC97L/Vector cells. In addition to MDR1, Shc3 modulated the expression of stemness-associated transcription factors, including OCT4, SOX2, and NANOG. These findings revealed that Shc3 is important for HCC stem cells expansion and that targeting Shc3 could be a promising strategy for HCC treatment.

Intrinsic or acquired MDR are major causes of chemoresistance, especially to doxorubicin. HCC chemoresistance is often attributed to overexpression of the MDR1 gene^29,30^, and MDR1 has been recognized as the driver of cancer multidrug resistance. Here, we revealed a novel finding that MDR1 expression is regulated by Shc3 via β-catenin/TCF pathway activation. Shc3 is significantly upregulated and its expression has a significantly positive correlation with MDR1 expression in HCC tumors. Our results further shown that Shc3 overexpression promotes MDR1 expression in HCC cells and decreases chemotherapeutic drug sensitivity. Additionally, inhibition of Shc3 expression could reduce MDR1 expression and increase drug sensitivity. This prominent correlation between the gene expression levels of Shc3 and MDR1 reveals that Shc3 may regulate MDR1 expression. However, the regulation of MDR1 expression remains poorly understood. Although recent reports have confirmed an upstream promoter region of MDR1 containing seven consensus binding sites of LEF/TCF in neuroblastoma, colorectal cancer, and breast cancer^26,31,32^, this regulation has not been investigated in HCC. As shown in Fig. 6C, β-catenin formed a complex with LEF/TCF at the binding site in the MDR1 promoter, establishing a direct connection between β-catenin and MDR1 in MHCC97L/Shc3 cells. Moreover, we found that Shc3 activated β-catenin/TCF pathway without Wnt stimulation, and knockdown of β-catenin significantly reduced the MDR1 expression and drug resistance ability induced by Shc3 overexpression. These findings demonstrate that the β-catenin/TCF signaling can be strongly activated by Shc3, and subsequently, can positively regulate MDR1 transcription in HCC cells. Moreover, this novel Shc3-dependent means to promote LEF/TCF activity and MDR1 expression represents a new molecular target for drug therapies.

Recent research has implied that the β-catenin/TCF signaling plays a key role in carcinogenesis by mediating cell chemoresistance and stem cell maintenance^33,34^. The “β-catenin destruction complex” and ubiquitin proteasome pathway regulated degradation of β-catenin are the central mechanism for the regulation of intracellular β-catenin levels^35^. Activation of the upstream signal transduction cascade leads to the disassociation of the destruction complex and suppresses phosphorylation and ubiquitin-proteasome-mediated degradation of β-catenin, which can enter the nucleus and then bind to the LEF/TCF transcription factors^36^. Aberrant β-catenin–LEF/TCF complex activation is frequently detected in human HCCs; hyper-activation of the β-catenin pathway mediates the expression of downstream target genes related to different cellular processes. Meanwhile, the elevated level of intracellular β-catenin expression correlated with shortened relapse-free survival, indicating the involvement of β-catenin activation in promoting recurrence of HCC^37^. Consequently, a better dissection of the mechanisms underlying the β-catenin/TCF pathway activation would improve HCC therapy effect. An important finding of this research was that Shc3 reduced the ubiquitination of β-catenin and activated β-catenin/TCF pathway in the absence of Wnt stimulation. First, we found that Shc3 interacted with the cytoplasmic components of β-catenin. Second, our results demonstrated that Shc3 is involved in the ubiquitin-proteasome-mediated degradation process of β-catenin and can function as a stabilizer. Knockdown of Shc3 promoted β-catenin ubiquitination, whereas overexpression of Shc3 significantly impaired the level of β-catenin ubiquitination. Shc3 is a member of the adaptor proteins that conserve domains with an identical PTB-CH1-SH2 domain. The PTB and SH2 domains are phospho-tyrosine-recognition modules that interact with tyrosine phosphorylated growth factor receptors, such as EGFR, RET, and TrkB (ref. ^38^). The CH1 and CH2 regions contain tyrosine-phosphorylation residues that recruit other adapter proteins, such as Grb2, MVP, and Grb2-associated binding protein 1 (Gab1)^10,11,39^. For the first time, our results reveal that Shc3 regulates β-catenin protein ubiquitination and degradation. We also found that β-catenin degradation by the destruction complex is destabilized by the binding between Shc3 and β-catenin, indicating that Shc3 mediated the β-catenin/TCF pathway in the cytoplasm, but not in the membrane where the Shc3 adaptor protein functions the best. These observations are consistent with previous studies that Shc3 was located in both the membrane and the cytoplasm. Therefore, this study verified the novel mechanism by which Shc3 independent Wnt activated β-catenin/TCF pathway. Thus, Shc3 promotes HCC recurrence via the potentiation of β-catenin signaling. This finding could partially explain the observation of high nuclear β-catenin in recurrent HCC compared to β-catenin.

In summary, we identified that Shc3 expression is significantly upregulated in HCC tumors and is strongly associated with MDR1/P-gp expression and HCC recurrence. We further reinforced the notion that Shc3 regulates MDR1 expression and reduces the sensitivity of HCC cells to sorafenib and doxorubicin treatment. First, Shc3 enhances stem cells maintenance by upregulating stemness-associated transcription factors, including SOX2, OCT4, and NANOG. Second, Shc3 interacts with β-catenin and inhibits destruction complex stability, promotes β-catenin release from the destruction complex, and dampens the β-catenin ubiquitination. Third, the Shc3-mediated β-catenin/TCF pathway can induce the activation of MDR1 and stemness-associated transcription factors, thus promoting HCC stemness and reducing sensitivity to sorafenib and doxorubicin. Moreover, we demonstrated that Shc3 can increase the drug resistance of HCC both in vitro and in vivo. The novel identified Shc3-β-catenin/TCF-MDR1 axis provides new insight into HCC MDR and represents a valuable target for HCC therapy.

## Materials and methods

### Cell lines and animals

Huh7 was purchased from the Japanese Collection of Research Biosources. HEK293T, PLC and HepG2 cells were purchased from the American Type Culture Collection biobank. MHCC97H, MHCC97L, and HCCLM3 cells were cultured as described^11^. All experiments used early-passage cells and cells were tested for Mycoplasma.

The xenograft mouse model was constructed by transplanting MHCC97L/Vector and MHCC97L/Shc3 cells subcutaneously to the nude mice. Animal care and handling procedures were performed in accordance with the guide for the care and use of laboratory animals, and the animal study protocol was approved by the institutional animal care and use committee of Tianjin Medical University.

### Clinical specimens

HCC tumor and adjacent para-tumor tissues from 52 patients were collected from September 2016 to December 2018 and the expression of mRNA level were detected by quantitative reverse-transcription PCR (qRT-PCR). Eight paired of HCC tissue samples were randomly selected from 52 HCC patients for western blot analysis (Table S1). What’s more, 72 paired paraffin-embedded HCC tissues were collected between 2017 and 2019 that used for immunohistochemistry (IHC). All clinical data collection and postoperative follow-up procedures were performed according to a uniform guideline, which were also recorded in Table 1. All human liver tissues were obtained from Henan Cancer Hospital Affiliated to Zhengzhou University. Informed consent was provided by patients, and ethics approval was obtained from Ethics Committee (No. 2016CT054) of Henan Cancer Hospital.

### Plasmids and transfection

Expression plasmids of p64Shc3 were constructed to pCDH-CMV-MCS lentivector (System Biosciences, America). Recombinant genes coding short hairpin RNAs (shRNA) against Shc3 were constructed to pLKO.1-TRC cloning vector (Addgene, America). The details of constructions of the expression plasmids or shRNA are described in Supplementary Materials and Methods (for shRNA sequences, see Table S2). β-catenin-siRNA duplexes and nontarget siRNA were designed and synthesized by RiboBio (China), and the sequences are listed in Table S3. Transient transfections were performed using Lipofectamine 3000 (Life Technologies, America). The primer sequences for qRT-PCR are listed in Table S4.

### Cellular uptake and intracellular location of doxorubicin

We observed the intracellular location of doxorubicin in MHCC97L cells by the confocal microscope. Cells were seeded on glass slides in 12-well culture plates at a density of 5 × 10^4^ cells/well and cultured for 24 h. Thereafter, cells were incubated separately with doxorubicin at a concentration of 1 μg/mL. At 4 h after incubation, cells were collected, fixed with 4% paraformaldehyde, and subsequently stained with DAPI for 10 min. Finally, all cells were mounted in DAKO mounting medium on glass slides and imaged using an FV-1000 confocal microscope (Olympus, Japan).

To investigate the amounts of doxorubicin uptake by MHCC97L cells using flow cytometry, cells were seeded in 6-well plates at a concentration of 2 × 10^5^ cells/well and allowed to attach for 24 h. After that, cells were incubated separately with doxorubicin concentration of 1 μg/mL. At 4 h after incubation, the cells were washed with phosphate-buffered saline (PBS) solution, digested with trypsin and resuspended in PBS solution. Finally, cell resuspensions were analyzed by a flow cytometer (BD FACSVerse, America).

### Tumor sphere formation assay

Cells (5000 cells per 1 mL) were cultured in ultra-low attachment 6-well plates in serum-free Dulbecco’s modified Eagle’s medium/F12 (Invitrogen, America) supplemented with B-27 (1:50; Invitrogen), 20 ng/mL epidermal growth factor (BD Biosciences, America), 20 ng/mL basic fibroblast growth factor (bFGF; BD Biosciences, America) and 10 ng/ml hepatocyte growth factor (PeproTech, America). Cells were cultured for more than 7 days and the cells were fed every 3 days.

### Immunoprecipitation (IP) and immunoblotting (IB) analysis

For coimmunoprecipitation, cell lysates were incubated on a rotator with antibody, at 4 °C overnight. The protein antibody protein A/G-dynabead complexes were prepared by adding 30 µL of protein A/G dynabeads (Life Technologies, America), a normal IgG control was assayed simultaneously. After centrifugation to pelletize the dynabeads, the supernatants were subjected to sodium dodecyl sulphate–polyacrylamide gel electrophoresis (SDS–PAGE) and IB. The immunocomplexes were analyzed by using liquid chromatography-tandem mass spectrometry (LC/MS-MS) or western blot analysis and these are performed as described previously^40^. The following antibodies were used in western blot analysis: P-gp (1:5000, ab170904,Abcam, UK), Shc3 (1:250, sc-365598, Santa Cruz Biotechnology, CA), GAPDH (1:500, sc-47724, Santa Cruz Biotechnology, CA), β-catenin (1:5000, ab32572, Abcam, UK), Axin (1:200, sc-293190, Santa Cruz Biotechnology, CA), GSK-3β (1:200, sc-377213, Santa Cruz Biotechnology, CA), H-3 (1:2000, ab1791, Abcam, UK), OCT4 (1:1000, #2750, Cell Signaling Technology, USA), SOX2 (1:1000, #3579, Cell Signaling Technology, USA).

### Ubiquitin ladder assay

Cell lysates were prepared by incubating cells in 1% Tris-Triton cell lysis buffer (Cell Signaling Technology, America) containing 1 mM phenylmethylsulfonyl fluoride (PMSF) and the protease inhibitor cocktail on ice for 30 min, following which, lysates were centrifuged at 12,000 × *g* for 10 min. The supernatants were incubated overnight with 30 μL Dynabeads Protein A (Life Technologies, America) precoated with anti-β-catenin (Abcam, Cambridge, UK), or anti-ubiquitin (Abcam, Cambridge, UK) antibodies. The immunocomplexes were analyzed by western blots. A normal IgG (Cell Signaling Technology, America) control was assayed simultaneously.

### Luciferase assay

Promoter reporters and dual-luciferase assay. MHCC97L cells were transfected with the TOP-FLASH and FOP-FLASH reporter plasmids (Addgene, America) together with pRL-TK. Luciferase activity was measured in a 1.5 mL Eppendorf tube with the Promega Dual-Luciferases Reporter Assay kit (Promega E1980, America) according to manufacturer’s protocols after transfection. Relative renilla luciferase activity was normalized to firefly luciferase activity.

### Xenografted liver cancer model in mice

Animal care and handling procedures were performed in accordance with the Guide for the Care and Use of Laboratory Animals, and the animal study protocol was approved by the Institutional Animal Care and Use Committee of Tianjin Medical University. The animals used to test the treatment were 5-week-old female BALB/c athymic nude mice. Briefly, 12 nude mice were randomly allocated to four groups and subcutaneously inoculated with the above MHCC97L/Vector and MHCC97L/Shc3 cells (2*10^6^ cells for each mouse, *n* = 3 mice per group) with different treatment. Two weeks later, the mice were treated with doxorubicin (20 μg/g) via intravenous injection every other day for consecutive four times, and the tumor volumes were monitored and recorded every three days. The investigator who monitored the tumor volumes was blinded to group allocation. The tumor volumes were estimated using the following formula: 0.5 × length × width^2^.

### Immunohistochemistry (IHC) staining

Shc3 (1:100,Santa Cruz Biotechnology, CA), P-gp (1:250, Abcam, UK) and β-catenin (1:500, Abcam, UK) were used for routine IHC stains on Dako EnVision two-step method in accordance with the manufacturer’s instructions.

### Statistical analysis

Clinicopathologic correlations were analyzed by Pearson’s *χ*^2^ test. The two-way ANOVA or Student *t*-test were used for comparison differences among three groups or between two groups, respectively. All statistical tests were two-sided, and differences were considered statistically significant for *P*-values less than 0.05. SPSS 22.0 software was used for all data analyzes.

## Supplementary information

Supplementary Figures

Supplementary Figure legends

Supplementary Table S1

Supplementary Table S2

Supplementary Table S3

Supplementary Table S4
