# Supplementary figures and images for: Shc3 promotes hepatocellular carcinoma stemness and drug resistance by interacting with β-catenin to inhibit its ubiquitin degradation pathway
Source: Cell Death Dis. 2021 Mar 15;12(3):278. doi: 10.1038/s41419-021-03560-8 (PMC7961052; doi:10.1038/s41419-021-03560-8)

**
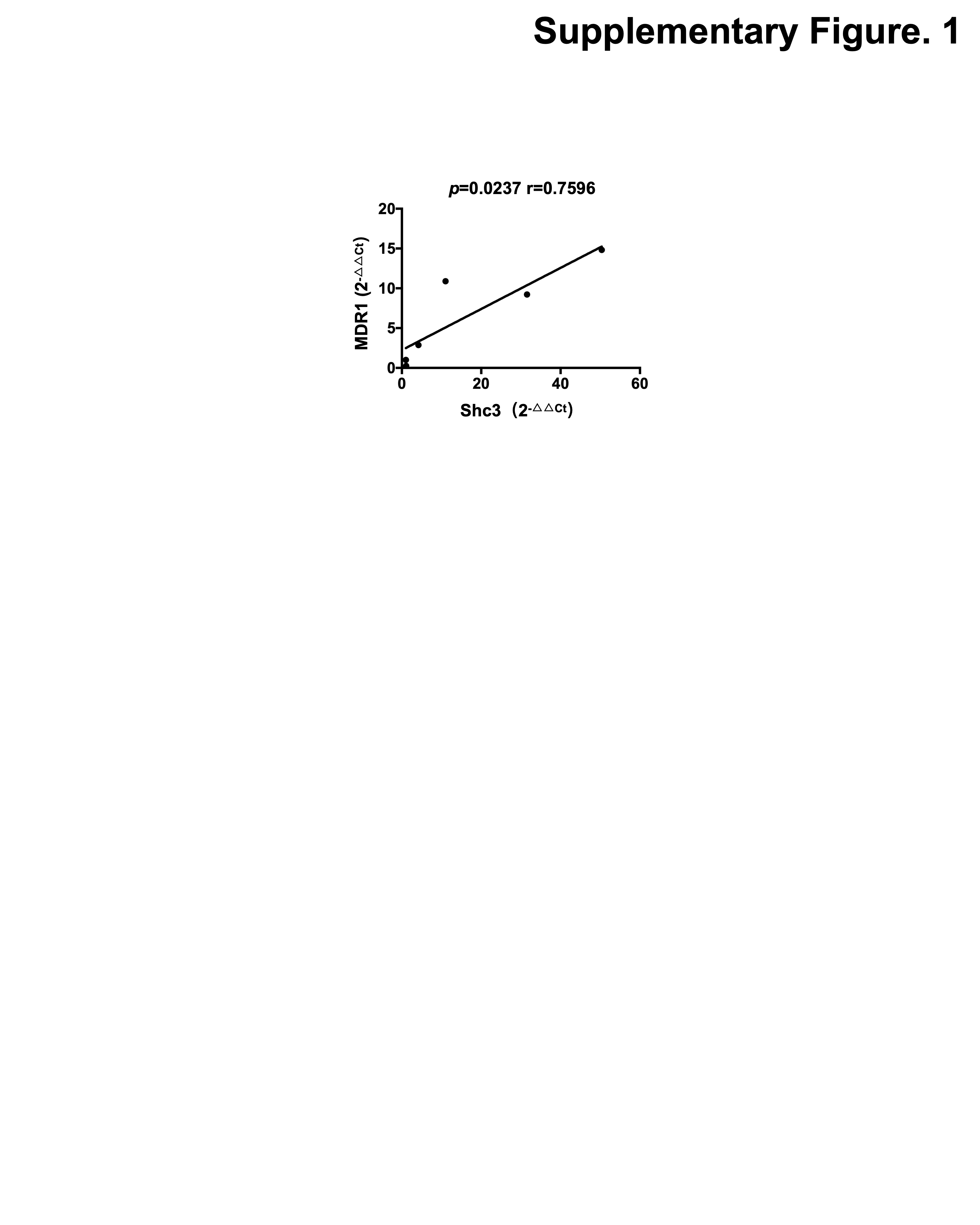
**

**
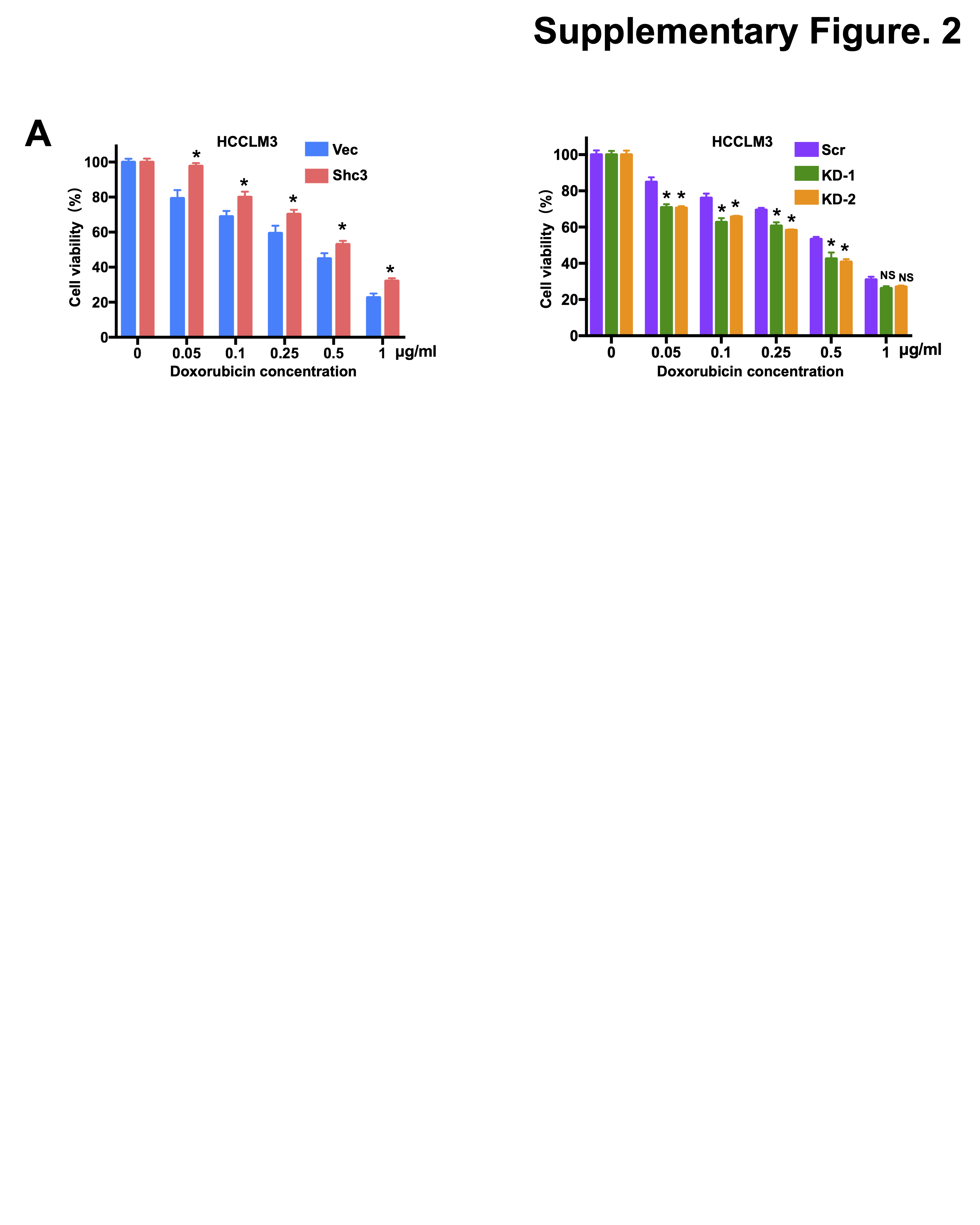
**

**
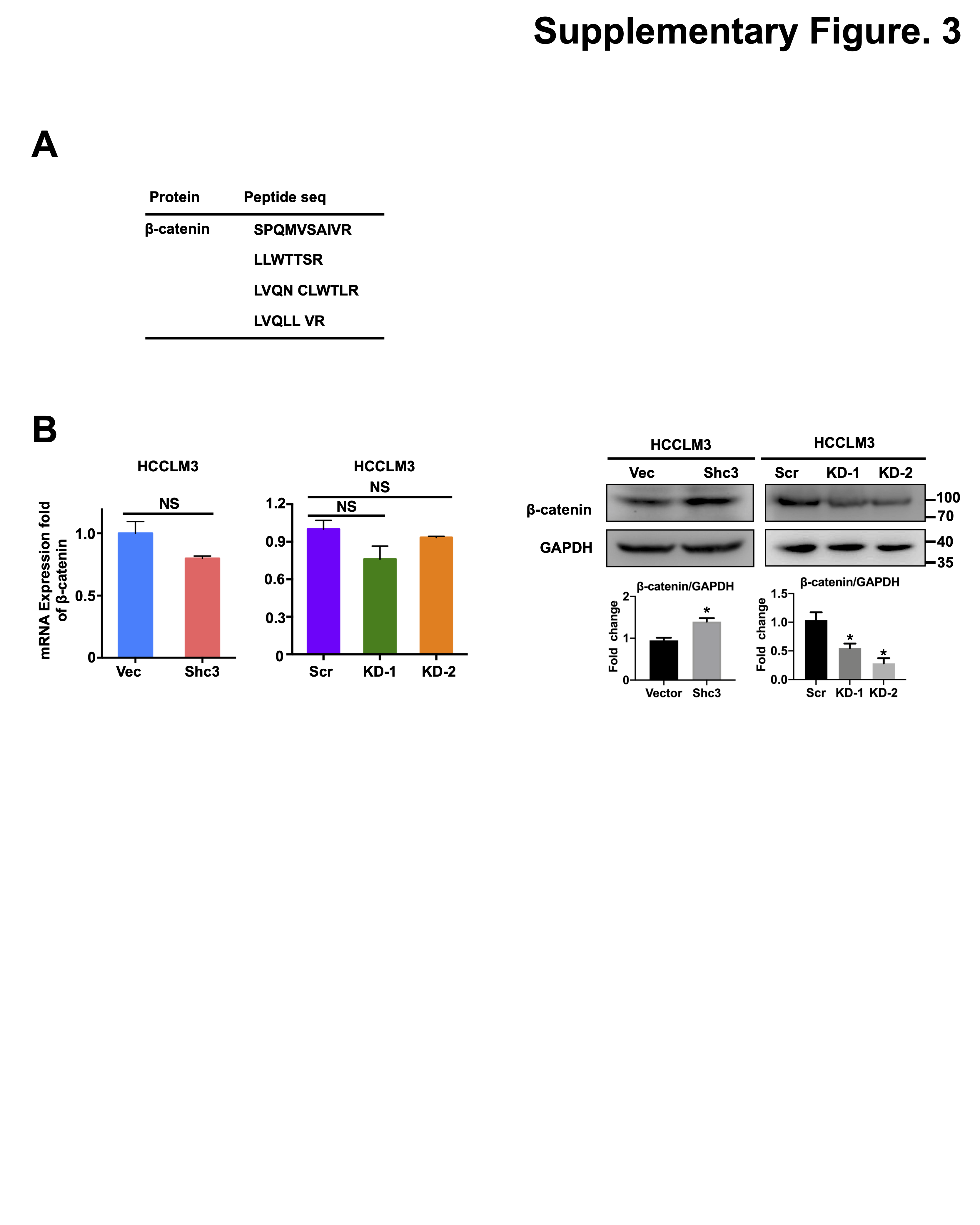
**

**
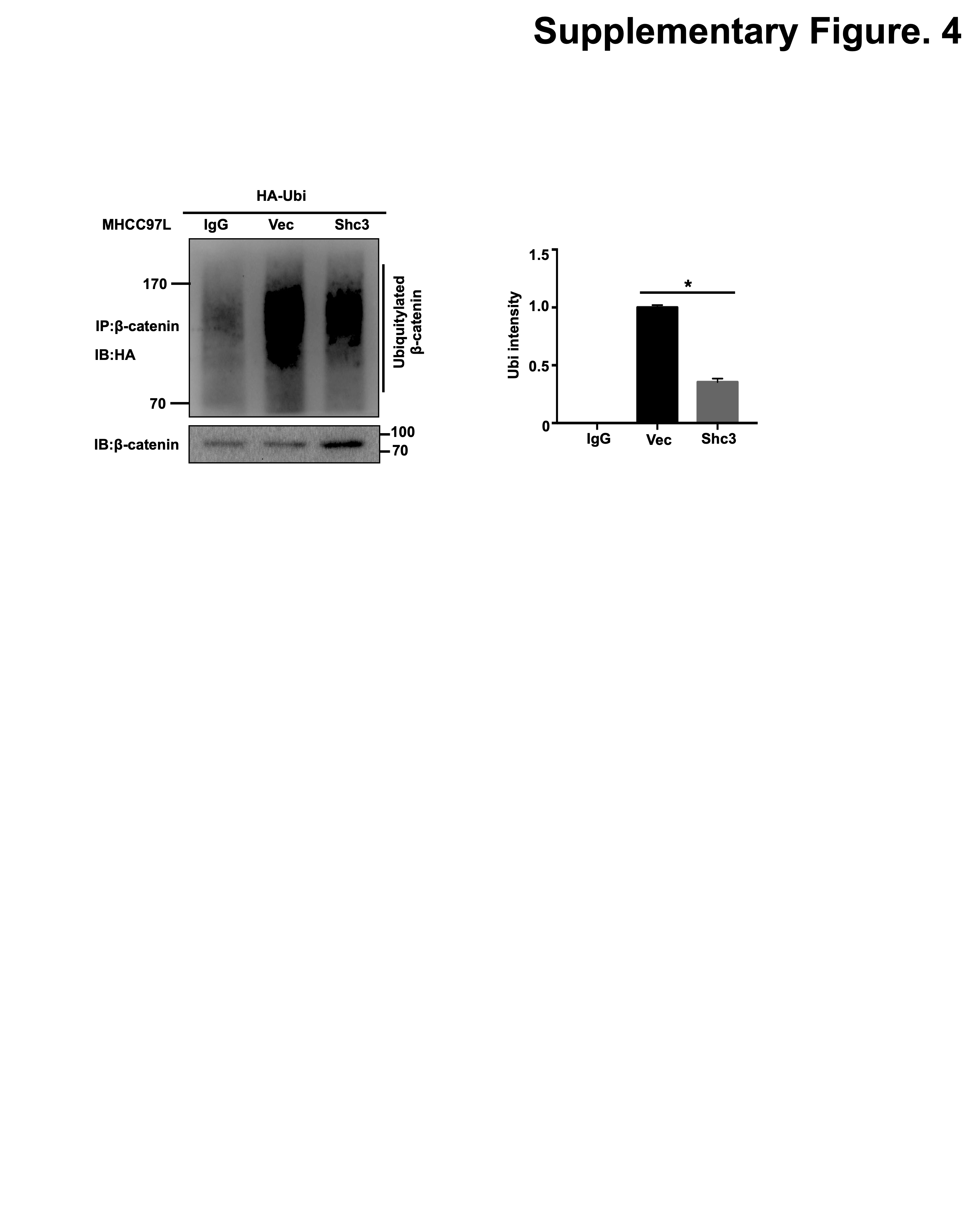
**

**
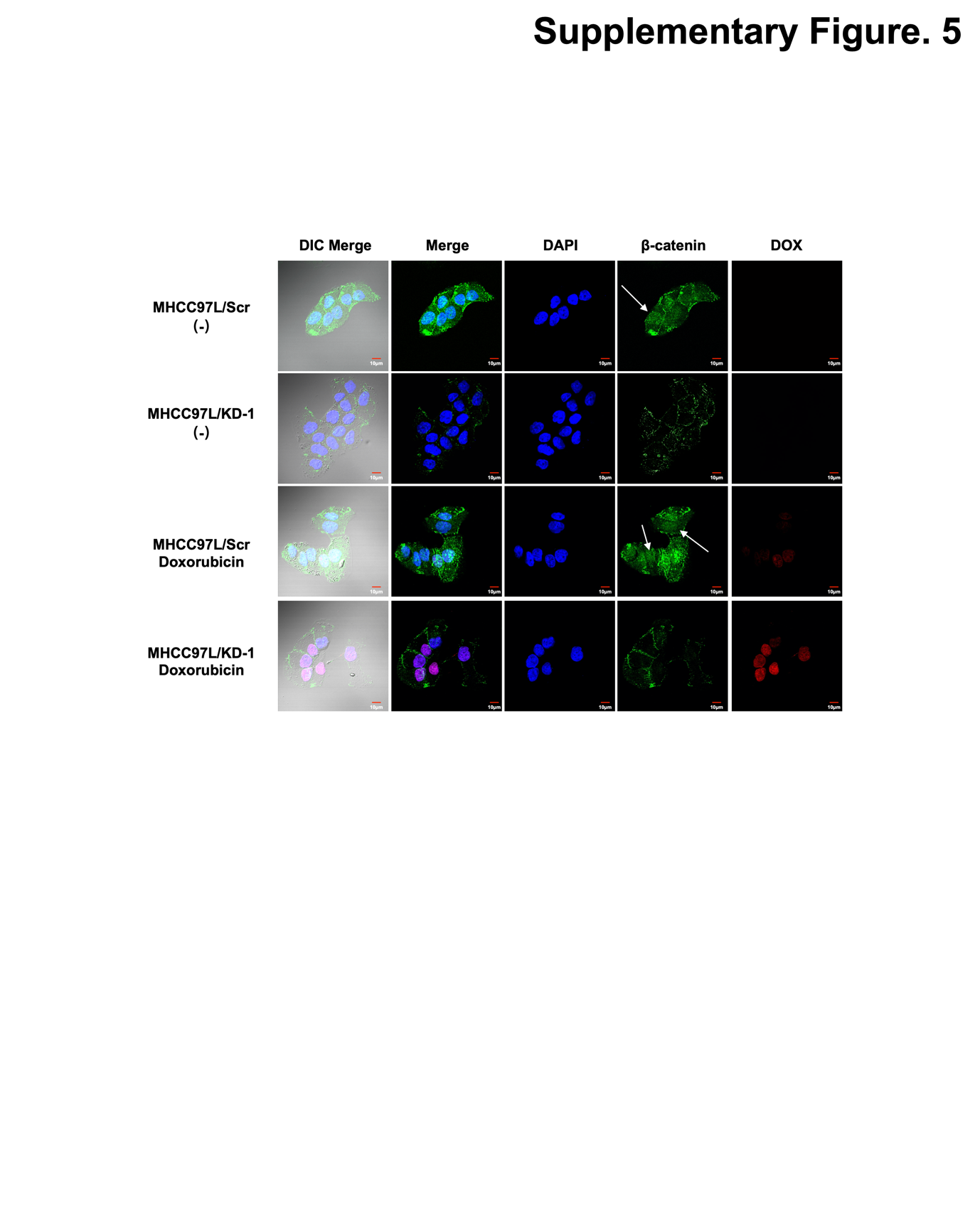
**

Supplement: Supplementary file 1 — Supplementary Figures [file 41419_2021_3560_MOESM1_ESM.doc]
