# Supplementary material for: Shc3 promotes hepatocellular carcinoma stemness and drug resistance by interacting with β-catenin to inhibit its ubiquitin degradation pathway
Source: Cell Death Dis. 2021 Mar 15;12(3):278. doi: 10.1038/s41419-021-03560-8 (PMC7961052; doi:10.1038/s41419-021-03560-8)
Supplement: Supplementary file 2 — Supplementary Figure legends [file 41419_2021_3560_MOESM2_ESM.doc]

**Supplementary Figure legends:**

**Figure S1.** The correlation between Shc3 and MDR1 mRNA expressions was analyzed in 6 HCC cell lines (Pearson’s correlation coefficient, *r* = 0.7596, **P* < 0.05).

**Figure S2.** A, Over-expression of Shc3 significantly promoted drug resistance of HCCLM3 cells to doxorubicin (left); Shc3 inhibition significantly restored the sensitivity of HCCLM3 cells to doxorubicin in a dose-dependent manner (right). HCCLM3 cells infected with lentiviruses expressing control or Shc3 short hairpin RNA were treated with drugs for 48 h. Cell viability was measured using the CCK8 assay and compared with the non-treated cells (100% viability). Data are shown as mean ± SD; n = 5; *, *P*<0.05 vs control, by Student’s *t*-test.

**Figure S3.** A, Peptide sequences of β-catenin interacting with Shc3 identified in MS. B, qRT-PCR and Western blot analysis of β-catenin expression levels in HCCLM3 Shc3-overexpressing and Shc3-knockdown cells. Right bottom, the histogram shows the comparison of the relative intensity of β-catenin and GAPDH. Data represent the means ± SD (*, *P* < 0.05, by Student *t* test).

**Figure S4.** After transfected with HA-Ubi overexpressing plasmid, the lysates of Shc3 overexpression cells and its control cells were prepared and immunoprecipitated with anti-β-catenin antibody, followed by Western blots with anti-HA (upper) or anti-β-catenin (lower). The ubiquitinated β-catenin bands are marked on the right. Data represent the means ± SD (*, *P* < 0.05, by Student *t* test).

**Figure S5.** IF staining of β-catenin (green), doxorubicin (red), and nuclei (DAPI, blue) in MHCC97L/Scr and MHCC97L/KD-1 cells that were treated or untreated with 1 μg/mL doxorubicin for 2 h. Representative images of three independent experiments are shown.

**Supplementary Tables**

**Table S1**. Clinicopathological information of the HCC tissue samples for qRT-PCR

**Table S2**. Sequences of the genes coding shRNA for Shc3 knockdown experiments

**Table S3**. Sequences of the genes coding siRNA for CTNNB1 knockdown experiments

**Table S4**. Sequences of the DNA primers for qRT-PCR
