# Supplementary material for: Shc3 promotes hepatocellular carcinoma stemness and drug resistance by interacting with β-catenin to inhibit its ubiquitin degradation pathway
Source: Cell Death Dis. 2021 Mar 15;12(3):278. doi: 10.1038/s41419-021-03560-8 (PMC7961052; doi:10.1038/s41419-021-03560-8)
Supplement: Supplementary file 3 — Supplementary Table S1 [file 41419_2021_3560_MOESM3_ESM.doc]

**Table S1**. Clinicopathological information of the HCC tissue samples for qRT-PCR

|  | All | Shc3 up-regulated | |
| --- | --- | --- | --- |
|  |  | Positive (n=43) | Negative (n=9) |
| Gender |  |  |  |
| Male | 40 | 34 | 6 |
| Female | 12 | 9 | 3 |
| Age |  |  |  |
| ≤60 years | 27 | 20 | 7 |
| >60 years | 25 | 23 | 2 |
| Serum AFP Level (ng/mL) |  |  |  |
| ≤400 | 37 | 31 | 6 |
| >400 | 15 | 12 | 3 |
| HBsAg |  |  |  |
| Positive | 39 | 33 | 6 |
| Negative | 13 | 10 | 3 |
| Maximal Tumor Size |  |  |  |
| ≤5cm | 23 | 18 | 5 |
| >5cm | 29 | 25 | 4 |
| Tumor Number |  |  |  |
| Single | 42 | 36 | 6 |
| Multiple | 10 | 7 | 3 |
