# Supplementary material for: Shc3 promotes hepatocellular carcinoma stemness and drug resistance by interacting with β-catenin to inhibit its ubiquitin degradation pathway
Source: Cell Death Dis. 2021 Mar 15;12(3):278. doi: 10.1038/s41419-021-03560-8 (PMC7961052; doi:10.1038/s41419-021-03560-8)
Supplement: Supplementary file 4 — Supplementary Table S2 [file 41419_2021_3560_MOESM4_ESM.doc]

**Table S2**. Sequences of the genes coding shRNA for Shc3 knockdown experiments

| Name | Target sequence |
| --- | --- |
| Shc3-KD1 | CTCCGGTTTAAGCAATATTTA |
| Shc3-KD2 | GCTCCGGTTTAAGCAATATTT |
| shControl | TCCTAAGGTTAAGTCGCCCTC |
