# Supplementary material for: Shc3 promotes hepatocellular carcinoma stemness and drug resistance by interacting with β-catenin to inhibit its ubiquitin degradation pathway
Source: Cell Death Dis. 2021 Mar 15;12(3):278. doi: 10.1038/s41419-021-03560-8 (PMC7961052; doi:10.1038/s41419-021-03560-8)
Supplement: Supplementary file 5 — Supplementary Table S3 [file 41419_2021_3560_MOESM5_ESM.doc]

**Table S3**. Sequences of the genes coding siRNA for CTNNB1 knockdown experiments

| Name | Target sequence |
| --- | --- |
| CTNNB1 | GGTGGTGGTTAATAAGGCT |
