# Supplementary material for: Shc3 promotes hepatocellular carcinoma stemness and drug resistance by interacting with β-catenin to inhibit its ubiquitin degradation pathway
Source: Cell Death Dis. 2021 Mar 15;12(3):278. doi: 10.1038/s41419-021-03560-8 (PMC7961052; doi:10.1038/s41419-021-03560-8)
Supplement: Supplementary file 6 — Supplementary Table S4 [file 41419_2021_3560_MOESM6_ESM.doc]

**Table S4**. Sequences of the DNA primers for qRT-PCR

| Name | Sequence (5’-3’) |
| --- | --- |
| Shc3 | Forward: AAGCCTTTGAGCTCCGGTTT  Reverse: GGAGGAGGCATCTTGCTTG |
| MDR1 | Forward: CCCATCATTGCAATAGCAGG  Reverse: GTTCAAACTTCTGCTCCTGA |
| GAPDH | Forward: TGCACCACCAACTGCTTAGC  Reverse: GGCATGGACTGTGGTCATGAG |
| CTNNB1 | Forward: AAGACATCACTGAGCCTGCCAT  Reverse: CGATTTGCGGGACAAAGGGCAA |
| NANOG | Forward: ACCTATGCCTGTGATTTGTGG  Reverse: AGTGGGTTGTTTGCCTTTGG |
| SOX2 | Forward: GGTTACCTCTTCCTCCCACTCC  Reverse: CCCTCCCATTTCCCTCGTTT |
| OCT4 | Forward: GAAAGCGAACCAGTATCGAGAAC  Reverse: CCCCTGAGAAAGGAGACCCA |
